# Late-Onset Mania in a Patient with Movement Disorder and Basal Ganglia Calcifications: A Challenge for Diagnosis and Treatment

**DOI:** 10.1155/2016/1393982

**Published:** 2016-04-24

**Authors:** Beatrice Roiter, Giorgio Pigato, Giulio Perugi

**Affiliations:** ^1^Department of Neurosciences, Section of Psychiatry, University of Padova, 35128 Padova, Italy; ^2^Department of Psychiatry, University of Pisa, 56121 Pisa, Italy; ^3^The Institute of Behavioral Sciences “G. De Lisio”, 56127 Pisa, Italy

## Abstract

Age of onset can have a significant impact on clinical course and pathophysiological mechanism of bipolar disorder. Late-onset bipolar episodes are more likely linked to medical illnesses and so are frequently classified as “secondary” forms of mood disorder. We discuss the case of a patient who at the age of 58 presented his first delusional-manic episode. He also had mild frontal and occipital cortical atrophy, white matter posterior ischemic lesions, and small basal ganglia calcifications. Seven years later, he presented a second manic episode with new emergent hyperkinetic choreiform symptoms. Taking into account movement disturbances, the presence of basal ganglia calcification, and worsening of cortical atrophy, we performed a differential diagnosis between Fahr disease, Fahr's syndrome, calcifications due to ageing, supersensitivity psychosis, and dementia. Valproate, quetiapine, and tetrabenazine were sequentially administered and yielded a good therapeutic response as regards manic and movement symptoms. Relationship between medications and course of specific symptoms was observed.

## 1. Introduction 

Bipolar disorder is reported to have onset prior to age of 50 in 90% of cases, with a peak age between 20 and 40 years. About 10% of cases are over the age of 50 years in their first episode and are usually referred to as “late-onset bipolar disorder” (LOBD) [1–3].

Late-onset bipolar symptomatology is often associated with medical conditions, mainly cerebrovascular disease or dementia. Interestingly, elderly patients with cognitive decline and mixed-labile manic state have been supposed to represent a less penetrant variant of bipolar disorder, defined as type VI [4].

We describe the case of a patient with basal ganglia calcifications who presented at the age of 58 years the first delirious-manic episode and at the age of 65 the second one, which was associated with hyperkinetic involuntary movements. He was finally diagnosed as LOBD since medical and neurological assessment were unremarkable. Ageing was the most probable etiology of basal ganglia calcifications.

Currently, there is lack of specific guidelines regarding pharmacological treatment of LOBD. Drugs with mood stabilizing properties are recommended [4, 5], while antidepressants are associated with worsening of symptoms. Therapies of movement and psychiatric symptoms associated with basal ganglia alterations are focused on symptomatic relief and evidences derived only from case reports or small case series [6–9].

## 2. Case Presentation 

Eight years ago a 58-year-old man was compulsorily admitted to our inpatient psychiatric unit because of a severe delirious-manic episode. He had no personal nor family psychiatric history although dysfunctional personality traits (rigidity, irritability, and impulsivity) were recognizable. He had no neurodevelopmental disorders, head trauma, surgery, infections, or exposure to toxic substances.

The manic episode was characterized by irritability, dysphoria, talkativeness, racing thoughts, hyperactivity, distractibility, grandiosity, persecutory delusions, psychomotor agitation, aggressiveness, insomnia, and mental confusion.

A complete medical history was obtained and clinical assessment confirmed long lasting thrombocytopenia, polyclonal gammopathy, hypertensive heart disease, and mild bilateral carotid stenosis. Other medical conditions were excluded and an extensive neuropsychological assessment (Mini Mental State Examination, Raven Coloured Progressive Matrices, Rey Auditory Verbal Learning Test, Semantic and Phonologic Verbal Fluency Tests, Boston Naming Test, Poppelreuter-Ghent's Overlapping Figures Test, and Barrage Testing) did not show any alterations. A brain computerized tomography (CT) scan showed mild diffuse cortical atrophy and small basal ganglia calcifications, but these findings were considered not clinically relevant. Valproate slow release (SR) was started up to 1500 mg/day and sequential add-on antipsychotic medications (haloperidol 12 mg/day, then olanzapine 20 mg/day) were unsuccessfully tried. Finally an antipsychotic switch to quetiapine SR (600 mg/day) yielded rapid and full symptomatic remission. The patient was discharged with a diagnosis of “bipolar disorder-manic episode.” The patient fully recovered returning to premorbid level of relational and personal functioning for the following seven years.

At the age of 65 the patient discontinued medications and relapsed with a delirious-manic episode associated with new emergent hyperkinetic movements. They were involuntary, generalized (involving face, trunk, and limbs), subcontinuous, jerky, choreic, and suppressible only for a few seconds. Behavioral symptoms also reappeared.

The patient was frequently confused, agitated, irritable, hyperactive (he moved abroad without any predefined scope and then came back complaining about jerky movements), and disinhibited (hyperconfidential). He also had visual hallucinations (seeing a woman holding gifts instead of a white sheet over a seat) and strange behavior (suddenly jumping to the ground and shouting for help, asking to eat only meat and honey). In addition he manifested distractibility, delirious episodes, confabulations, insomnia, and poor insight. These symptoms worsened in the evening. The patient also had weight loss and manifested episodes of sudden falls, loss of consciousness, and sphincter release. He was repeatedly visited in emergency room by psychiatrists and neurologists and diagnoses were “tardive dyskinesia,” “psychogenic movements disorders,” “psychosis,” and “dementia.” He was also briefly hospitalized twice in a neurology ward. Further examinations (electroencephalography [EEG], cerebral CT, magnetic resonance imaging [MRI], angio-MRI, neurocognitive tests, and CT-positron emission tomography [PET] total body) were carried out without significant alterations. Cognitive tests were the same used during the first episode and showed deficits of verbal material learning only.

**Figure 1:**
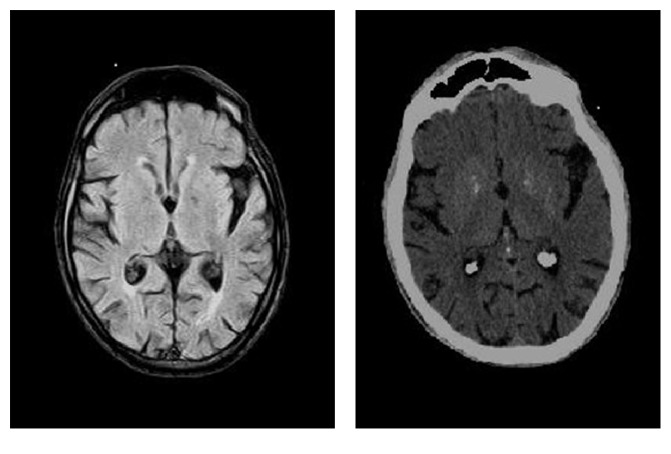
MRI scan on the left and CT scan on the right performed under pharmacological sedation in order to reduce motion artifacts, showing moderate diffuse cortical atrophy and small bilateral basal ganglia calcifications.

Finally the patient was admitted to our psychiatric unit. A brain CT scan and a brain MRI were repeated with pharmacological sedation. They showed moderate diffuse cortical atrophy, especially in frontal and occipital regions; posterior white matter lesions due to chronic vascular ischemia; and small bilateral pallidal calcifications (Figure 1). Brain ^18^[F]fludeoxyglucose PET confirmed a diffuse cortical hypometabolism. We performed further extensive medical examinations which excluded phosphocalcium metabolism alterations, tumors, and infections.

Pharmacological treatment was performed as follows. Firstly, valproate acid SR (1200 mg/day) and quetiapine SR (800 mg/day) were started with good response for behavioral-mood symptoms. Secondly, tetrabenazine was added (up to 75 mg/day) and motor disturbances significantly ameliorated. Furthermore, we noted that every time our patient refused to take medications symptoms markedly and suddenly (<12 hours) worsened especially with reappearance of hyperkinetic movements. The patient recovered and regained partial personal and relational functioning. He was discharged and agreed to admission to a geriatric nursing institute.

## 3. Discussion

This case report presents interesting challenges regarding diagnosis and pharmacotherapy since the patient showed his first episode at the age of 58 and the second one seven years later associated with new emergent hyperkinetic movements.

“Severe delirious-manic episode,” “LOBD,” “bipolar type VI,” and “bipolar type pseudodementia” are descriptive terms and they refer to possible expressions of bipolar spectrum. Patients with LOBD present bipolar diathesis which can be elicited by medical conditions (i.e., vitamin B12 deficiency, hyperthyroidism, Cushing's syndrome, and corticosteroid administration) or by many neurologic conditions such as Alzheimer's disease, frontotemporal dementia, vascular dementia, silent cerebral infarcts and stroke, normal pressure hydrocephalus, brain tumors, brain injury, epilepsy, infections of the central nervous system, Huntington's disease, and prion diseases [10–14]. Interestingly some authors described, as clinical variant of LOBD, a bipolar type VI disorder, which is characterized by mixed-labile mood symptoms and cognitive dysfunction associated with hyperthymic/cyclothymic/irritable temperament, family history of bipolar disorder, refractoriness to antidepressants, and acetylcholinesterase inhibitors but favourable response to mood stabilizers and/or atypical antipsychotics [4]. In our case no underlying medical or neurological causes were found, so the diagnosis of delirium was excluded. Diagnosis of dementia was also excluded because frontal atrophy was judged not clinically relevant; cognitive alterations were fluctuating and reversible and cognitive tests were not significantly altered. Thus, the cognitive dysfunctions were considered as manifestations of bipolar type pseudodementia.

Basal ganglia calcifications (BGC) also have been associated with manic-psychotic symptoms [15–20] and can be linked to some specific different conditions, that is, Fahr disease (FD), Fahr's syndrome (FS), and ageing. At the patient's second episode, we observed again manic and delirious symptoms plus new emergent hyperkinetic choreiform movements. We detected a worsening of cerebral atrophy but neurocognitive assessment, showing normal findings with the only exception of verbal learning deficits, excluded a diagnosis of dementia. We therefore performed an extensive clinical assessment of basal ganglia calcifications.

We believed that FD was not probable because typical features described in the literature were lacking, such as family history for movement disorders [15, 17] or intracranial calcifications in putamen, caudate nucleus, dentate nucleus, thalamus, and cerebellum [21]. We could not perform genetic tests [22], but the patient's DNA was stored for future analysis. We excluded FS because the most frequent causes of intracranial calcifications such as phosphocalcium metabolism alterations, tumors, and infections [23–25] were not found. Thus we interpretated ageing to be the most likely condition giving rise to basal ganglia calcifications in the patient. The extant literature states that in the elderly the calcifications are usually small and their degrees do not always correlate with severity of symptoms [24, 26]. We also ruled out the diagnosis of Huntington's disease, since there was no caudate atrophy and geneticists did not consider it clinically justified to perform DNA testing.

Our patient significantly improved with psychotropic polypharmacy. Particularly, we observed that behavioral, mood, and psychotic symptoms responded to valproate and quetiapine. This combination was chosen because it had yielded good response in the previous episode. There is lack of knowledge about acute treatment of mania in elderly bipolar patients. Lithium and valproate are the most recommended drugs while the role of atypical antipsychotics needs to be clarified [5, 27]. The patient's first manic episode responded only after quetiapine was added on to valproate. This questions the manic-like features because it is impossible to know whether valproate had any real impact. However quetiapine is recognized as antimanic agent especially among elderly bipolar patients with delirious features and high doses of both valproate and quetiapine are not effective in the treatment of delirium. Mood stabilizers and/or second-generation antipsychotics were beneficial also in psychiatric symptoms associated with BGC [7–9].

Hyperkinetic symptoms improved with tetrabenazine, which we administered on empirical basis [28]. We also noted that when the patient refused medications both psychobehavioral and hyperkinetic symptoms reappeared.

Taken together, our case was diagnosed with bipolar disorder type VI because we detected some of its typical features, such as premorbid irritable-hyperthymic temperament, delirious-manic episodes, and good response to mood stabilizer/atypical antipsychotics. Furthermore, we supposed that the pathophysiology of the manic and hyperkinetic symptoms could have been partly explained by basal ganglia calcifications. The progressive worsening of frontal atrophy could have disrupted key-circuits involved in inhibitory control and modulation of movement, mood, and behavior.

With regard to the second episode, psychotic, delirious, and motor symptoms might additionally be at least partially explained by “supersensitivity” phenomena [29–31]. Supersensitivity psychosis (SSP) refers to a rapid onset psychotic symptomatology emerging during an adequate compliance to high potency antipsychotics or after withdrawal of short half-lives antipsychotics. SSP is supposed to be linked to long-term antipsychotic administration and consequent dopamine D2-receptors-supersensitivity adaptation in the mesolimbic pathways, rather than to the reemergence of underlying illness. This mechanism is analogous to nigrostriatal dopamine supersensitivity involved in tardive dyskinesia, which often cooccurs.

Our patient presented features suggestive of SSP, such as rapid onset of psychotic symptoms after drug withdrawal, occurrence of involuntary movements, absence of major life events, and regression of motor symptoms with antipsychotics and tetrabenazine. In our case the antipsychotic was a short half-life low-potency medication, while SSP is more frequently described with high potency antipsychotics and other short half-lives antipsychotics such as clozapine [32]. In any case it has been suggested that patients with bipolar disorder are more prone to develop supersensitivity and tardive movement disorders after long-term antipsychotic exposure [33]. Furthermore, the presence of basal ganglia calcifications and frontal atrophy may have also favoured the development of SSP. This case report underscores the relevance of neurological investigations in patients with LOBD and the complex interaction between bipolar disorder diathesis, neurocognitive decline, and pharmacological treatments with antipsychotics.
